# Negotiating Shanghai Mercy Hospital: Philanthropy, Business and Control of Madness in Republican China

**DOI:** 10.1093/shm/hkad019

**Published:** 2023-05-13

**Authors:** Jinping Ma

**Affiliations:** Department of History, Xiamen University, Daxue Rd, Siming District, Xiamen, Fujian, China, 361005

**Keywords:** psychopathic hospital, medical financing, colonial medicine, China modernisation, transnational politicking

## Abstract

This study examines the initiation and administration of Mercy Hospital in Republican Shanghai. It explains the protracted negotiations that underpinned the collaboration between the Chinese founder Lu Bohong, the Shanghai Municipal Council (SMC) of the International Settlement and the Municipal Administration (FMA) of the French Concession. Despite mutual needs for a psychiatric hospital, the collaboration was undermined by disputes over funding shares and administrative direction. While Lu expected a symbolic modern philanthropy, the SMC and FMA saw it as an economic tool to relieve the responsibility of regulating refugees and the increasing mental patients. They repeatedly forced Lu to make concessions with financial instruments, but ended up non-cooperation, leading the patrons to compromise to keep their problem solver. However, after Lu’s murder and the subsequent dysfunction of the Chinese municipal government, the SMC and FMA could not help but take on this task to protect their settlements from the threat.

Asylums and mental hospitals were significant components of colonialism in the East, Southeast Asia and Australia.^1^ The appropriation of their establishment and administration complemented colonial control and manifested national modernity. The institutionalisation of mental health care in Republican China is the focus of recent groundbreaking research on medicine and modernity. One direction emphasised the indigenisation of scientific knowledge (such as psychology, psychiatry and neurology) to the East, including the reinterpretation and appropriation of ‘mental illness’ in trans-cultural practices, and the representation of indigenous concepts of mental problems such as *feng*, *dian* and *kuang*.^2^

Another direction, inspired in part by Foucault’s anthropological research, is oriented towards the socio-political approach, which focuses on the institutionalisation and engagement of various actors with political, financial and professional interests.^3^ In the case of the John Kerr Refuge in Canton, the first insane asylum in China, missionaries deliberately left out the socio-historical and political conditions of home care; thus, partially supporting the shift of responsibility for public safety to the private sector and allowing the police to confine some mental patients to the asylums.^4^ Its maintenance depended on an agreement between the governments of Canton and Hong Kong on the repatriation of patients to Hong Kong, Canton and Britain.^5^ By comparison, the construction of the Beijing Psychopathic Hospital was the result of cooperation and compromise in funding, administration and supervision of treatment between Beijing authorities, including the Social Affairs Bureau, the Ministry of Hygiene and the Peking Union Medical College (PUMC), a school modelled after Johns Hopkins University School of Medicine, which was taken over by the Rockefeller Foundation in 1915.^6^

Multi-governance and -funding characterised the development of biomedicine in early twentieth-century China. Heretofore, medical agents of missionaries and colonial authorities had been able to operate freely due to China’s weak diplomacy. The waves of anti-imperialist and nationalistic movements since the late 1890s forced Westerners to cede more power to the Chinese, who now came to the fore. Crucial to the functioning of medical organisations work were the efforts to raise the money and resources.^7^ Chinese reformers tried all sorts of methods to raise money, from Chinese and foreign governmental and non-governmental organisations to members of the Chinese diaspora. The resulting complex composition of sponsors caused much discord and friction, with management and budget control being the main problems.

Mercy Hospital for Nervous Diseases (hereafter Mercy Hospital) exemplified hospital politics in a particular urban setting in Shanghai. It was founded on 29 June 1935 by Lu Bohong (Joseph Lo Pa Hong, Loh Pa-hung 陆伯鸿, 1875–1937), its initiator, and two subscribers, the Shanghai Municipal Council (SMC) and the French Municipal Administration (FMA). Lu was a well-known Catholic entrepreneur and philanthropist in Shanghai who gained fame for his business acumen, his extensive connections with both the Chinese gentry and foreign powers, and his charity in the fields of medicine, education and religion.^8^ Lu’s fame reached its peak when he was elected chairman of Catholic Action at its first national congress in September 1935, 3 months after Mercy Hospital opened. Figure 1 shows this moment with Lu in the full regalia of the Knight of Colombus. Mercy Hospital was one of his most famous achievements. The SMC and FMA were administrative bodies of the International Settlement and French Concession, which almost marginalised the Chinese-controlled districts. They shaped the development of modern Shanghai in all areas, the most important of which were public health and policing. The SMC had always been active in municipal administration, while the FMA chose this project because it relied on Lu’s local network to maintain political relations and business stability. Moreover, both were in urgent need of an adequate solution for the increasing number of mentally ill people. Therefore, Lu’s plan was greeted with joy. However, their cooperation was marked by constant tensions and compromises.

**Fig. 1:**
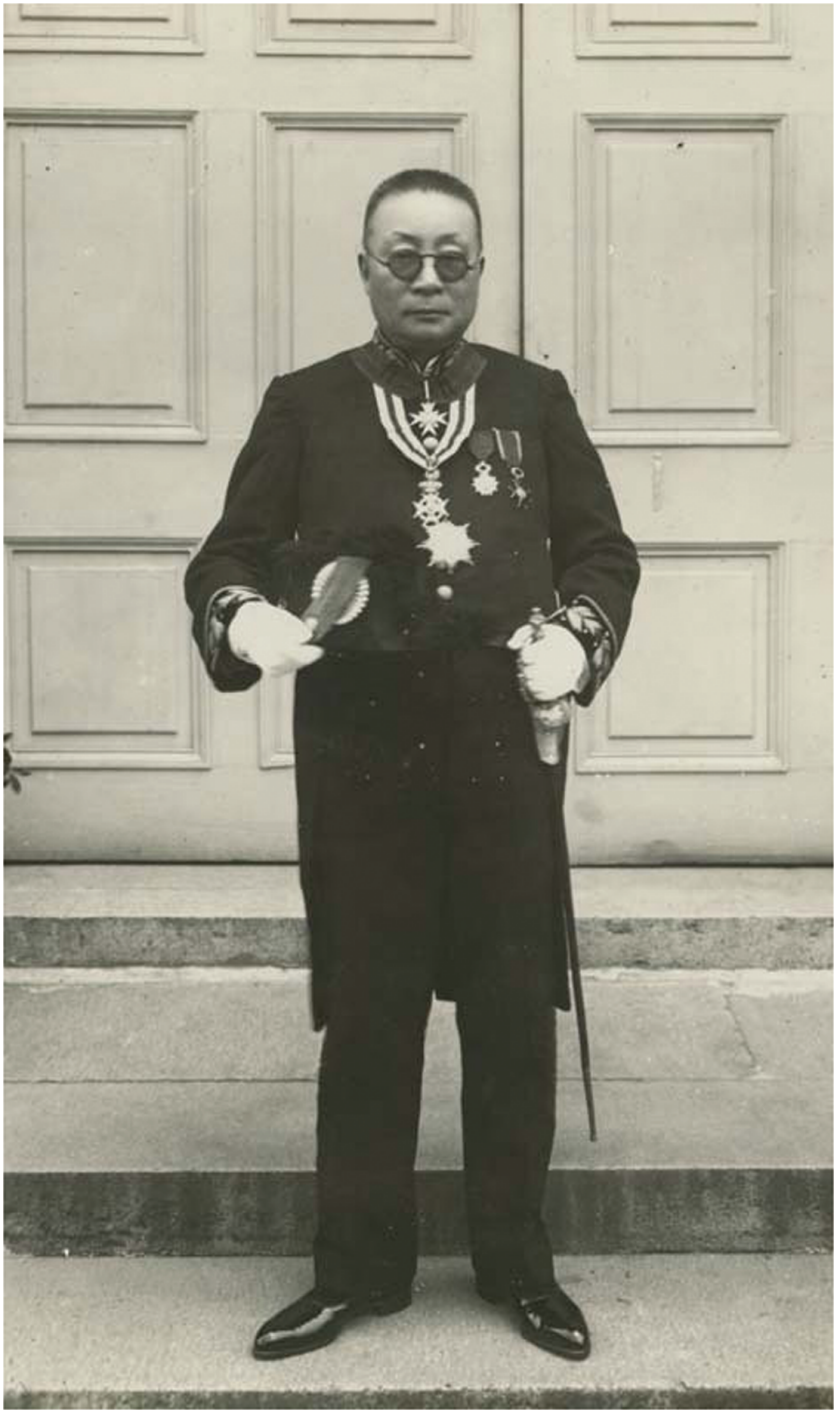
Lo Pahong in full Knight of Colombus regalia. Whitworth University (2017). Album 13: Catholic Action First National Congress in Shanghai, 1935. Paper 93. https://digitalcommons.whitworth.edu/album13/93

This study seeks to deepen the transnational politicking approach and illustrate the mechanism of hospital financing by analysing the operation of Mercy Hospital. It will show how competing powers and interest groups appropriated this hospital as a symbol of modernity and an instrument of racial politics and diplomacy to reduce tensions and strengthen control. Wen-ji Wang points to the impact of transnational cooperation on the emergence of knowledge and services in the field of mental hygiene.^9^ However, this study will focus on the behind-the-scenes negotiations of the different actors around the daily operation of the hospital. It will analyse the friction between Lu’s philanthropic vision and SMC’s business orientation, which resulted from the competing goals of expanding aid against economic confinement and racial superiority.^10^ I argue that both orientations prioritised improving treatment, but also compromised to achieve cooperation.

This article’s first two sections will examine how Mercy Hospital was founded in response to diverse needs and promoted as a symbol of a modern, professional organisation. I refer mainly to reports from *Shen Bao* (申报), the longest lasting and arguably most significant newspaper in modern China, particularly concerning legal reform and all-around coverage of the opening ceremony of the Mercy Hospital. These reports were mostly selective but complementary to piece together a complete social image of the hospital. The third section will unravel the especially complex urban setting of Shanghai and even more intricate relations among the three sponsors. I will explain their standpoints and motivations, which gave rise to later disputes over hospital management. The remaining three parts will chronically dissect the three stages of Mercy Hospital from 1935 to the foundation of the PRC, according to changes to its guidelines and features. The principal sources are underused SMC records, especially correspondence within and among the patrons and minutes of council meetings, supplemented with archives from the hospital itself. These reliable sources provide a thorough account of Mercy Hospital’s unique history.

## Call for Institutional Mental Health Care

From the second half of the nineteenth century, the Qing government undertook structural self-strengthening and westernisation to catch up with the modernised states. However, defeat in the First Sino-Japanese War marked the failure of half a century of reformation and deeply humiliated the Chinese. Later nationalist movements overthrew the Qing monarchy along with its holistic reforms, many of which were radical.

Politically, a new republican regime was created to emulate the ‘advanced states’ of Europe, America and Japan. Social security for citizens and building a united nation according to the norms of science were important criteria for governance and necessary for entering into negotiations with foreign powers in exchange for extraterritoriality. Institutional confinement, including prisons and asylums, was a prestigious symbol of progressive nations and became a political priority for legal reform from the early twentieth century.^11^ Republican regimes programmed, actively or passively, the regulation of the mentally disabled as an important part of constabulary management.

Culturally, the entire Chinese moral and value system was called into question. China was ‘diagnosed’ as the ‘sick man of East Asia’, both the biological and the social body.^12^ Medical science was introduced and social engineering was launched to restore the national vitality. In contrast, to the doctrine of genetic determinism, the modernising intellectuals viewed heredity as a flexible process that could be improved through human interventions such as education and discipline.^13^ The modernising elites sought to monitor, correct and cultivate the everyday behaviours and mentalities of people, especially children, through programmes such as the New Life Movements and the Mental Hygiene Movements. All aimed at better conforming to the image of a modern nation. Thus, those who exhibited deviant behaviour such as mental illness or ideological nonconformity were ‘classified as mentally ill—and consequently became the targets of professional intervention’.^14^

The call for psychopathic institutions stems from both internal reforms and external pressures. China has a long history of legal control of the insane. Vivien Ng describes how the mentally ill were regulated after 1731 through compulsory registration and confinement at home, and detention in gaols where family supervision was impossible.^15^ In the late nineteenth century, home confinement was increasingly criticised by missionaries stationed in China, who stressed that the insane were largely unprovided for and treated inhumanely, for example by being chained. Qing diplomats attending international conferences also faced pressure to integrate global standards into China, including developing mental therapy.^16^ Japan’s successful Meiji Restoration provided a good model of non-white modernity for China’s reforms at the turn of the century. One important aspect was the extension of state power into the lives and bodies of citizens through the regulation of hygiene and mental health. While Japanese institutionalisation of mental service combined public and private hospitals with traditional practices, including home confinement, Chinese reformers were more oriented towards the Western example.^17^ After visiting prisons, asylums and hospitals in Japan, the Minister of Law Shen Jiaben (沈家本, 1840–1913) proposed imprisoning the condemned insane.^18^ But the seriously mentally ill offenders were to be housed in psychiatric hospitals and not in prison because, on the one hand, they could not serve their sentences and, on the other, the prison had to pay for their treatment. This concept was also intended to prevent seriously ill people from committing minor offences in order to receive free treatment in prison.^19^

The inauguration of Mercy came against the background of earlier experiences with mental institutions/hospitals in cities that dealt with foreigners, such as Guangzhou, Shanghai, Beijing and Suzhou. However, no other city could match Shanghai in the number and complexity of mental institutions.^20^ From 1907, both the International and French concessions funded wards, such as the Russian Orthodox Confraternity Hospital and a Mental Ward in the Victoria Nursing Home attached to the General Hospital to temporarily treat and confine mentally ill foreigners. Although there was a push to repatriate most of the patients as soon as possible, the departments were still overcrowded in 1926 and needed urgent expansion in 1929.^21^

Chinese doctors ran private hospitals to meet the needs of Chinese patients. The *Zhongguo fengbing yiyuan* (China Hospital for the Insane 中国疯病医院) was founded in 1911 and operated until 1934.^22^ In 1933, the Chinese Medicine practitioner Gu Wenjun (顾文俊) opened another short-lived hospital, the *Shanghai fengdian zhuanmen yiyuan* (Shanghai Specialised Hospital for the Insane上海疯癫专门医院, 1933–36).^23^ Both advertised an untested high success rate, quick discharge and various therapies. They also catered to burgeoning nationalism by promoting the presence and efficacy—or myth—of ‘native’ doctors, traditional prescriptions and techniques such as elixir, acupuncture or diagnosis by *shouzhen* (hand observation 手诊).

Public charities also tried to separate the mad from other sheltered individuals. One of these was the St Joseph Hospice (上海新普育堂*Shanghai* New *Puyütang*, Shanghai Hsing Piu Yoh Tong, 1914), which combined Chinese charity hall with Catholic influences dating back to its founder, Lu Bohong, and chose to build a wing for the mentally ill.^24^ This was an economic decision driven by budget constraints and police requirements. Local police precincts were responsible for sending committing vagrants and the mentally ill to almshouses or detention. Charities shared the task of welfare with the local government in exchange for annual grants.

Both the Chinese and Concessions expected a larger hospital to alleviate the increasing overcrowding. The number of unidentifiable patients arriving with the refugees skyrocketed in the 1920s and 1930s as warfare intensified in China and Europe. The councils of two foreign Concessions found it difficult to avoid their responsibilities, as the police were tasked with removing insane offenders from the Settlements. Before Mercy, they had funded the Chinese Hospital for the Insane to remove patients from the hospitals within the Settlements.^25^

Meanwhile, Chinese educators called for a professional hospital for neuropsychiatric research and teaching. Neuropsychiatry was not institutionally integrated into the municipal apparatus until the early Nanjing Nationalist Government period (1927–37) when patriotic Chinese intellectuals set out to build Chinese-run institutions as an alternative to missionary and imperial organisations. PUMC, the most prestigious missionary university funded by the Rockefeller Foundation, was the exact counterpart. Yan Fuqing (颜福庆, 1882–1970), a former employee of the PUMC who left because of the unequal pay between Chinese and foreign faculties, founded the National Shanghai Medical College in Shanghai, which was the centre of the New Culture movements. Mental health problems were of particular importance because they symbolised China’s ‘mental backwardness’. Yan therefore planned to build a specialised hospital for neurotics in Jiangwan (Kiangwan 江湾), a suburb of Shanghai controlled by the Chinese authorities. His expectations were later fulfilled by Lu Bohong, who agreed to exchange the hospital facilities for medical support.

The Mercy Hospital in Shanghai was thus part of the call for nationalist reformation, combined with the institutional need to enlarge asylum space and relocate the insane, and the educational need for professional medical training. It was expected to demonstrate China’s progress and modernisation, taken on the role of charities, and meet the accommodation needs of foreign Concessions. These different expectations led to serious conflicts during the construction of Mercy Hospital.

## Mercy Hospital: A Showcase of Modernity

On 29 June 1935, the Mercy Hospital for Nervous Diseases in Beiqiao, a suburb of Shanghai, was inaugurated by three central figures: Lu Bohong, Fanny Halpern (the medical director) and the Reverend Father Schulz of the Steyl Mission. The ceremony was attended by more than three hundred dignitaries, with the mayor’s representative giving the opening speech and ending with comments from the head of Shanghai county. All major media covered the inauguration ceremony.

Mercy Hospital was referred to as China’s ‘first psychiatric hospital’, referring to its status as the first hospital founded and run by Chinese.^26^ The earlier hospitals employed Chinese staff, but Mercy was distinguished by the fact that its founder was Chinese. Lu ownership, but not foreign ownership, was thus crucial to maintaining national status despite the various patrons. This emphasis was in line with the prevailing nationalism and showed that China met a criterion of modernity and progress—a response to the adage that ‘all modern municipalities provide an asylum for the insane’.^27^ As the Mayor’s representative pointed out, Mercy Hospital reflected the concern of the Chinese municipal government about civic hygiene and the end of previously ‘inhumane’ treatments of neuropathy.^28^ Consequently, Mercy became a symbolic political site for Chinese and European politicians who visited the hospital to see for themselves its uniqueness, grandeur, specialisation and popularity in East Asia.^29^

Mercy Hospital’s achievement was evident in its grand scale. Lu raised a fund of 500,000 *yuan*, of which 250,000 came from the public (especially local elites and Catholic Action), 100,000 from the Shanghai City Government, 100,000 from the SMC and 50,000 from the FMA. He bought more than 24 acres of land and constructed 16 buildings with a capacity of 600 beds.^30^ The numbers vary in different reports, but all praise the large scale. The design and furnishings followed the standard of the large sanatoria in Austria, and were equipped with telephones and electricity.^31^ Much of the equipment was taken from the world-leading mental institutes in Vienna.^32^

Medical equipment was central to its professional image. A team of foreign specialists was recruited and directed by Fanny Gisela Halpern, a psychiatrist who had graduated from the leading medical school at the University of Vienna. She was assisted by German and American Catholic brothers and sisters who worked as nurses, 12 for each sex. The division of wards was carefully thought out for both sexes. Violent patients were housed in specially designed wards walled with fine spring mattresses and floored with fine rubber. Patients could even choose different theme colours.^33^ Patients were admitted by walk-in or appointment, and family members could visit or even stay on site.^34^ Patients were treated with the latest cures such as hydrotherapy and occupational therapy, electro-shock therapy and surgical treatment. Part of the hospital was used as a medical university to train local staff.^35^

Halpern played a key role not only in the construction and maintenance of Mercy Hospital, but also in promoting mental hygiene in Shanghai. Because of her outstanding experience working with world-class psychiatrists in Vienna, she was invited to lead the development of psychiatry and neurology at the National Shanghai Medical College in 1933. Her professionalism was the most important criterion in convincing Lu of the need for a psychiatric hospital in Shanghai and establishing Mercy Hospital’s leading position in China, comparable to the Beijing Psychopathic Hospital. For example, at the inauguration ceremony, she declared that mental disorders were curable. Mercy Hospital was a ‘real’ modern hospital in every sense, especially with its scientifically trained medical staff who conformed to sound principles of modern psychiatry.^36^ But she placed more emphasis on prevention than cure, and found that there was a lack of social workers and local psychiatrists in China.^37^ Thus, she worked with other universities and missionaries to form a Mental Hygiene Association and promote the Mental Hygiene Movement. Mercy Hospital was an important focal point for these activities.

The name of the sanatorium was chosen specifically to allay any misgivings or shame about sending family members to an asylum or a psychopathic hospital.^38^ In Chinese, it was named *Puci Liaoyangyuan* (Puci Sanatorium, 普慈疗养院), meaning the Universal Mercy Sanatorium. Considering the shame of ‘backwardness’ associated with mental deficits, this naming reassured the mentally ill and their families that their social standing would remain intact. Commercially, it attracted patients seeking recuperation and convalescence.^39^ Located in a suburb, it was ideal for recovery with its secluded setting, fresh air, spacious gardens and seclusion from urban noise. The prospect of paying patients was an significant factor in these designs.

**Fig. 2:**
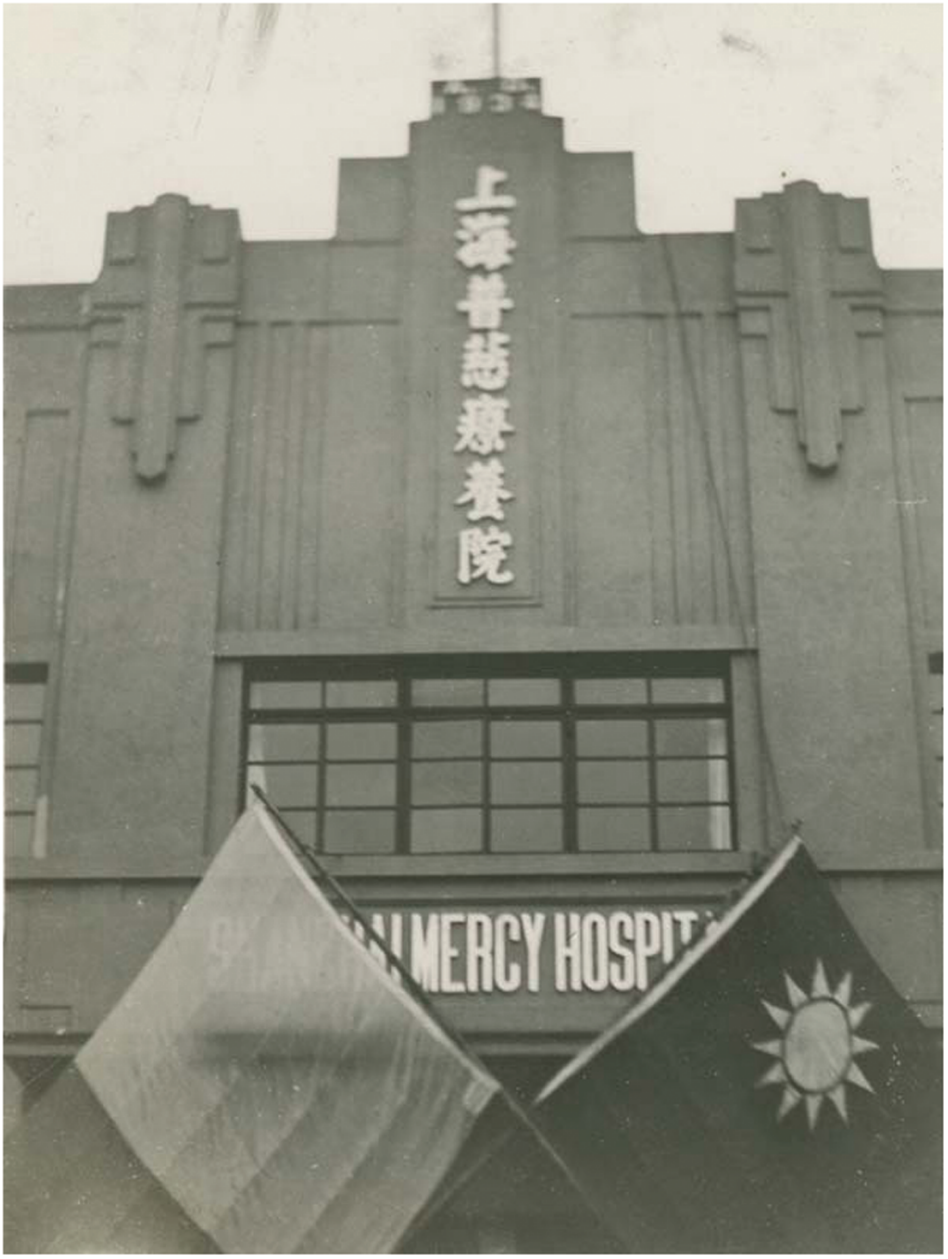
Shanghai Mercy Hospital 1. Whitworth University (2017). Album 13: Catholic Action First National Congress in Shanghai, 1935. Paper 1. https://digitalcommons.whitworth.edu/album13/1

Religion was an essential part of the hospital. The Buddhist word *puci* suggests a trans-cultural feature of benevolence. Such an image of charity was aimed at both Chinese and foreigners. It was in line with local charity halls such as the preceding New *puyütang* (universal feeding hall) and the universalism of Catholic missionaries. It also helped to absorb donations from government agencies and organisations. Nevertheless, the essential aims lay in its beneficence to indigent patients.^40^ Missionary influence was evident in the Catholicism of Lu and the bishop, Catholic Action funding and Catholic caregivers. Patients and deceased were baptised. Visiting Mercy Hospital was on the programme of the First National Congress of Catholic Action.^41^Figure 2 show this visit at the entrance door of Mercy Hospital with flags of Catholic Action (left) and the Republic of China (right).

Although the Mercy Hospital was portrayed as a grand project well received by the authorities and the populace, the stories behind the scenes were more complicated. The composition of the patrons and their conflicts with the local gentry were a constant source of annoyance. Lu encountered numerous obstacles in raising funds from the Concessions, who repeatedly fought for more say in the budget and management control. At times, Mercy Hospital was seen more as a negotiator in the containment of undesirables than as a hospital for the needs of the mentally ill. The next section will first unravel the complicated relationships between the actors before analysing their protracted negotiations.

## Shanghai Complexity and Competing Actors

After opening up to foreign trade in November 1843 under the Treaty of Nanjing, which ended the first Anglo-Chinese Opium War, Shanghai’s history under colonial control of the century began. Numerous foreign powers, most of them British, came to amass fortunes through colonial rule and extraterritoriality. They all established their national settlements first and were hungry for expansion. By 1930, Shanghai had risen to become the fifth largest city in the world and was synonymous with ‘modernity’ and all things flashy, fast and sophisticated.^42^ It served as the primary base for political, economic, military and new cultural revolutions, as well as a laboratory and stage for further education and trans-cultural exchange. For this reason, all clandestine or conspicuous forces made the city their base or competed for control of its resources. Waves of travellers, missionaries and refugees also passed through the city in search of new opportunities.

**Chart 1:**
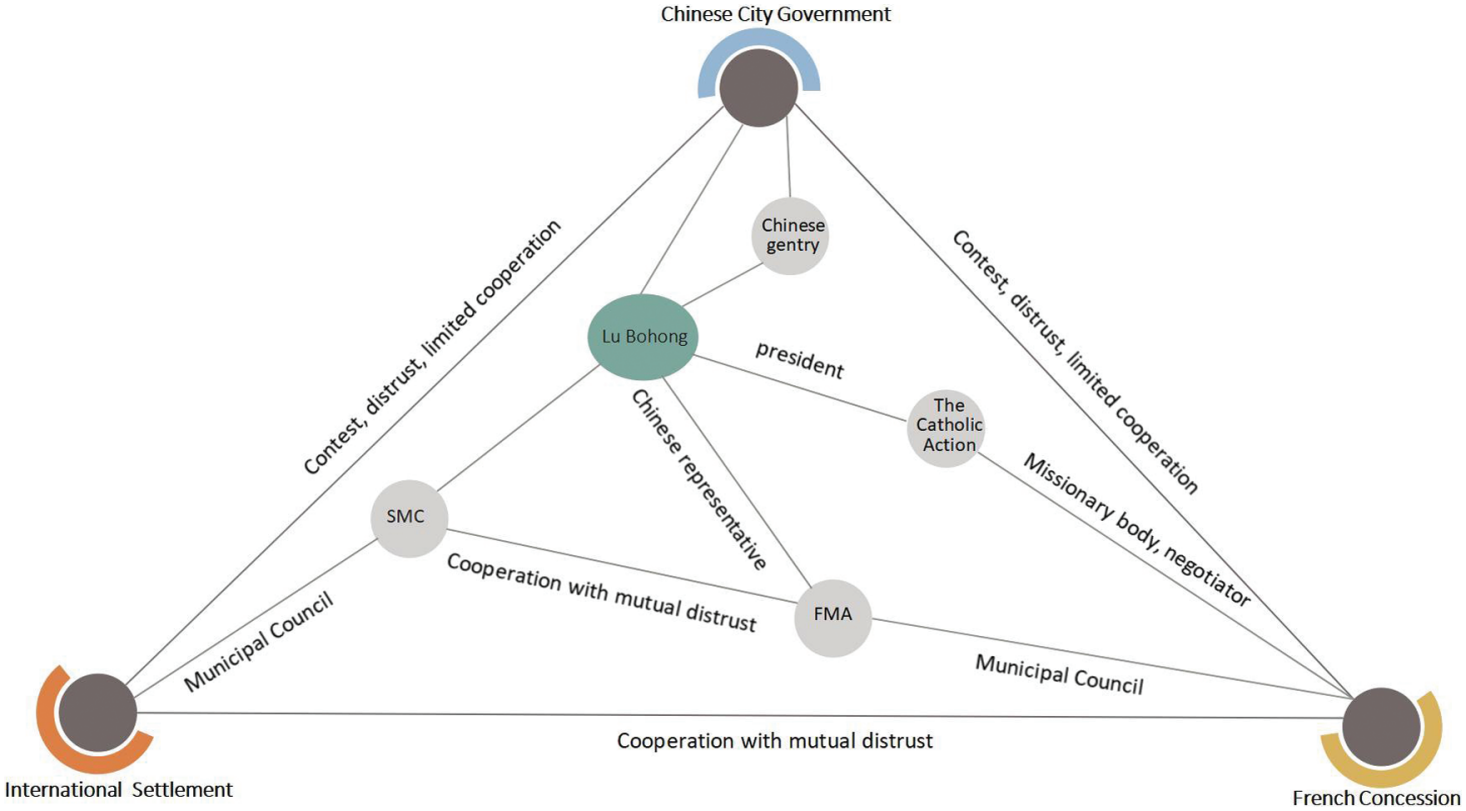
Relationships of competing agencies and actors

The city was under the control of many powers, but was mainly dominated by three settlements: the International Settlement, the French Concession and the marginalised Chinese city. This complexity meant that almost no project could be completed without the cooperation of other parties and interests. And cooperation was never easy, because the missions, goals and values were different or contradictory. Chart 1 generalises the complicated relationships between agencies and actors. The next few pages will analyse these relationships in detail.

The International Settlement was an 1863 merger of the British and American enclaves with several other trading powers, including the Germans, Russians and Japanese, joined in after they established treaty relations with China. It was governed by the SMC, a unique transnational and colonial institution that exercised wide-ranging governmental power dominated by British influences. However, the SMC, which behaved like an aggressive and militarised city-state, was independent of the imperial and native governments and subject to the ever-changing foreign community.^43^ This hybrid form of colonial city government was referred to by Jackson as ‘transnational colonialism’.^44^

The local French community initially advocated joining the International Settlement, but Paris refused to concede control of the Concession. Thus, the French Concession remained separate and formally became part of the French Empire under the authority of the Governor General of Indochina. ‘The Consul-General acted like a colonial governor with the *Conseil municipal* merely as an advisory role’.^45^

Both councils went to great lengths to maintain the supremacy and superiority of the ‘whites’. On the one hand, they set bars on the representation of Chinese elites and capitalists in the Council, segregated Chinese residents and policed the refugees. Municipal employees were also divided by ‘a strict racial hierarchy, with Britons occupying senior posts, Chinese undertaking the vast majority of the work, and other nationals operating in between’.^46^ The compromise could only be reached under enormous pressure from the anti-imperialist movements or in the face of anticipated business interests. On the other hand, the Councils avoided the presence of ‘defective human material’ by, for example, sending back chronically mentally ill foreigners and regulating white prostitutes.^47^ Racial segregation also worked for the insane. All ‘Orientals’, including the Japanese, were excluded from the mental ward in the early days. Later, however, they had to admit the growing number of white ‘mentally deficient who are Shanghai-born’.^48^

The SMC primarily served the interests of business and functioned like a corporate board. This character increasingly came into play as the importance of International Settlement increased in global trade, finance and investment. The concern for public health also had its origins in protecting employees and workers from disease and was always intended to be cost-effective. Because of the settlers’ preoccupation with business and trade, the SMC was governed by a ‘board of directors, with a chairman elected from among its members to lead meetings and represent the SMC’.^49^ British merchants dominated the ‘board’ throughout its existence.

Although both Settlements were unwilling to spend money beyond the basic needs for a safe living and working environment for foreign residents, SMC developed better public health management. International Settlement developed rapidly in the latter half of the nineteenth century, along with the increase in population. The Council had to introduce preventive medicine to protect the lives and health of the foreign residents. In addition, public health management became a service that a modern government was expected to provide.^50^ The SMC initially appointed a single health officer. But it soon evolved into a separate Public Health Department at the turn of the century, which established urban isolation hospitals to combat infectious diseases.^51^

The expansion of Public Health Department had a significant impact on the development of public health in Shanghai, but it was not meant for the poor. Foreigners without money would be repatriated at the expense of their consulate or national societies, while Chinese would be picked up by the police and extradited. Every effort was made to have those who fell out of the above two categories taken care of by charitable institutions, both to save costs and to maintain public order.^52^ This strategy was increasingly difficult to enforce as the stateless, poor and non-white population grew. Thus, limited welfare and charity for the refugees was established as complement to policing. The Mercy Hospital project was one such example.

The relationship between the FMA and the SMC has been a troubled one, characterised by cooperation on respective needs and frequent hesitation, haggling or conflict over services with no guarantee of equal funding. Competitive imperialism played a role. Although its health service was built as a last resort, the SMC tried to burnish its reputation for good governance, especially in comparison with the French.^53^ This superiority increased communal spending. As a purely colonial extension organisation, the FMA preferred to pay for the SMC’s medical services rather than establish a comprehensive communal health system.^54^ Conflicts arose in dealing with the increasingly troublesome people. The mutual distrust and frequent boundary violations by the residents led to both sides complaining about taking greater share and treating patients from each other’s settlements.^55^

One of the most controversial areas was the treatment of the mentally ill, which in many cases was long-term and unsupervised, and exceeded the capacity of the wards and the Council’s budget. More importantly, the incessant stream of Chinese mental patients, indigents and homeless being sent away and then returned angered the SMC, which joined with the FMA to put pressure on the Chinese authorities to share ‘municipal governing responsibilities’.^56^

The Chinese authorities entered the field of public health in 1927 and experienced strained relations with the Public Health Department. At this time, the new Special Municipality of Greater Shanghai (later called Shanghai City Government) was established to contest and control the authority of the foreign settlements and prepare to replace them. It drew up an ambitious plan of infrastructure and social welfare with hygiene central to its modernity and legitimacy. However, as a newly established city government, it was weak in finance and inexperienced in public health. Therefore, cooperation with foreign parties was the only way. However, the public health arena was exploited for political mistrust and usually hindered bilateral willingness to compromise on controversies. The Chinese Municipality’s modernity was also discounted or dropped at times of difficulty or urgency.

In the case of Mercy Hospital, the multiple and conflicting identities of its founder, Lu Bohong, further complicated the situation. First, as one of the first five Chinese members of the FMA, he was a mediator for the councils and a representative for the Chinese. When Mercy Hospital was inaugurated, the anti-imperial Chinese were still actively fighting for actual representation in both councils. The trilateral negotiations over control of the hospital thus paralleled the struggles against white supremacy.

Second, Lu’s religious and philanthropic goals were at odds with the Council’s business approaches. Lu had long been committed to medical charity and had earned great renown for it.^57^ Mercy Hospital was his next major philanthropy plan, which meant he would go all out to invest money and secure control. This is doomed to conflict with SMC’s business orientation and superiority in leadership.

Third, Lu’s contribution to Catholic missions defused his clash with the FMA. His conversion into a determined Catholic benefactor was one of the most successful achievements of the Jiangnan Mission. Under the tides of anti-imperialist and criticism of the unequal treatment of European and Chinese priests, Catholic associations were finding it increasingly difficult to recruit priests from the Chinese middle and upper classes.^58^ It was Lu who provided the impetus for a lay apostolic movement, founding and presiding over the Catholic Action Society in 1911 to ‘nurture a more devout religious life, to evangelise and to alleviate the suffering of the poor’.^59^ Although the FMA and the French consulate viewed Catholic missions as complementary means of providing important social services, they still needed them to ensure social and political stability, especially in times of crisis.^60^ Lu was such a figure of translator, negotiator and liaison. And for this reason, he was able to gain the support of Chinese officials and funding from Chinese and Western businessmen, from whom he gained wealth, influence and a seat in the city parliament.^61^

Fourth, Lu Bohong had every reason to seek glory and power rather than bow to the Concessions.^62^ Although foreign Christians believed Lu’s charity stemmed from his Christian identity, Lu had actually struggled for fame and power in his charitable endeavours before his murder by anti-Christian activists in 1937.^63^ This motivation, coupled with his energetic and steadfast personality, led to ongoing difficulties with the concession authorities, as explored in this paper.

Mental health was one of the most contentious issues in colonial Shanghai. The SMC and Lu were the main adversaries in the Mercy Hospital case. The FMA was not active in the urban investment, the missionaries took over the welfare part and Lu acted as the representative of Catholic Actions. While the SMC demanded cooperation with and an equal share from the FMA, the latter could often choose to cooperate with the SMC or side with the Catholics and Lu. Although the Chinese community was willing to shoulder its responsibilities, it often could not make ends meet. As an initiator and fundraiser, Lu was as much concerned with taking control of the hospital as with charity, which sometimes clashed with SMC’s business-oriented goals. However, their multilateral relations and conflicts were far more complicated than that, as the following section shows.

## Benevolence for the Lunatic

The complexity behind Mercy’s grandeur is primarily attributed to other expectations beyond the dispute over medical and charitable functions. The real need for a larger asylum facilitated their collaboration, but SMC’s colonial hegemony and cultural superiority and Lu’s resistance proved to be obstacles to smooth progress. Such conflicts were triggered by the first deficit and used to negotiate control of the hospital. The real needs of the patients were never the primary concern of either side. However, their conflicts arose before the initiation of Mercy Hospital.

The original initiative for Mercy Hospital was to alleviate overcrowding at St Joseph Hospice, which was housing large numbers of homeless and mentally ill people. Consideration had been given to building two rooms for male and female mental patients. But in December 1933, Lu changed his mind and drew up an ambitious plan for two asylums with an achievable budget of 300,000 *yuan*.^64^ To raise money, he submitted his proposal to the SMC for financial assistance.

Mercy Hospital offered an ideal solution to the SMC’s dilemma of housing the insane outside the foreign Concessions. As mentioned earlier, the SMC and the FMA faced increasing pressure from the growing influx of indigents.^65^ They preferred to deport foreign subjects to consular bodies with a 60-day moratorium, while Chinese persons were referred home or to charities by the police or the court. Concessional Councils tried to keep as few patients as possible in their settlements. Plans to build new asylums were repeatedly rejected when mental wards faced the urgent question of whether to discharge patients and expand space for the increasing number of chronic long-term patients in the 1920s. The Councils feared that increased capacity would lead to a deliberate influx from other precincts and put greater pressure on their administration.

Not surprisingly, J. H. Jordan, SMC’s third Commissioner of Public Health and a key figure in the Mercy Hospital case, was enthusiastic about Lu’s proposal. Jordan was an upper-class professional whom the SMC had carefully selected and recruited directly from Britain. His father had been a British minister in Beijing. Jordan studied at Cambridge before becoming a major in the Royal Army Medical Corps. He was considered a capable worker and quickly proved to be so. Jordan’s starting salary at the SMC was 30% higher than that of his predecessor.^66^ The reason Jordan took this position may have been to conduct colonial medical research, to make a name for himself in international academia and to advance professionally.^67^ Jordan differed from his predecessors’ approach to economic administration.^68^ He was passionate about conducting medical research, using the latest medicine in public health, publishing in the best journals and raising the international prestige of the Public Health Department. During his tenure, both the Public Health Department’s expenditure and its share of the city’s expenditure increased steadily. It is therefore not surprising that Jordan actively participated in negotiations within the SMC and between the two foreign Councils and successfully secured an increase in allocations for his department. Mercy Hospital was one of the priorities during his tenure.

The supposed cooperation was hampered by distrust of Chinese standards. Jordan judged Lu’s plan, although touted as modern, as very sketchy and failing to meet many criteria.^69^ For example, the wooden roof and floors were poorly designed in terms of fire safety in light of a British case where ‘the fire at Colney Hatch Asylum (London) in the wing for Jewish women burnt fifty-two of the inmates to death’.^70^ Jordan also opposed the congregated housing of violent offenders, although Lu believed that this design would cause the least disruption to mild patients.^71^ Nevertheless, Lu was willing to make changes in line with Jordan’s demands. For example, he promised to build padded cells and a separate house for foreign cases, and to divide new buildings into three sections to avoid fires.

Jordan’s discontent embodied white supremacy and cultural prejudice. In his letter to the SMC Board, he described rice as an unsuitable food for European patients. If given occasionally, he opined, ‘it could represent a pleasant change of diet; however, as a steady institutional diet, it was not unlike the bread and water given to obdurate prisoners in ancient days’, which be detrimental to their mental condition, and subsequent recovery.^72^ The higher medical standards demanded helped defend their pride in medical progress. There was also a fear that mistreatment would lead to lower ratings by patients’ families and that they would refuse to send patients to Mercy in the future.^73^

Despite the dissatisfaction, Mercy Hospital was still considered the best economic choice for housing patients and was worthy of Jordan’s lobbying. At a meeting on 6 February 1934, the Public Health Department agreed to support the construction of Mercy if the other two adjoining municipalities would ‘adopt a similar course’.^74^ In return, the SMC demanded free beds, building inspections and satisfactory conditions. This decision was conditional on considerations to reduce expenditure. As Jordan reiterated, the grants to Mercy ‘prove(d) far more economical than would the building and running of (a new) Medical Institutions under municipal standards’, which could have cost at least twice as much.^75^ Despite its unsatisfactory size, Mercy contributed to ‘clear up the unsatisfactory position of the Council in relation to lunatics amongst the Chinese population’.^76^ These benefits justified the funding for Mercy Hospital. Besides, this expenditure could be as frugal as possible if Mercy Hospital were fully utilised.

The inadequacies were thus easily forgiven, considering that the hospital was built on the cheapest criteria. Therefore, Jordan did not propose at SMC meetings to ‘discuss its deficiencies as seen from the standpoint of a modern Mental Hospital of this sort abroad’.^77^ On the contrary, he concluded that Mercy Hospital’s achievements were impressive. In the meanwhile, he urged Lu to make improvements, like finishing the heating system, before it admitted patients.^78^

The SMC had also cautiously assessed the price-quality ratio of investment. Before Mercy, it had funded two mental wards, the Russian Orthodox Confraternity Hospital and the Municipal Mental Ward, the latter to be expanded for expansion. In comparison, funding Mercy meant an estimated saving of 50,000 *yuan*. However, this saving would be more than eaten up by the approval of Lu’s application for a further grant of 82,000 *yuan*.^79^ When the Russian Orthodox Confraternity Hospital took the opportunity to apply for continuous funding, the SMC agreed to fund it as a backup plan.^80^

Most of the time, the SMC made decisions based on responses from the FMA and the Chinese City Government. If follow-up funding could be agreed internally, they usually waited until other municipalities approved grants. For example, the assessment of the annual grant was postponed from 1936 to 1937 to take advantage of the FMA’s actions and to ensure that the grant was calculated less arbitrarily. Lu also used this interdependence to achieve his fundraising goals. Upon receiving funds from the FMA and the Chinese City Government, he immediately assessed the added value of the SMC in order to obtain grants, which was often successful.^81^

With the first major deficit in December 1935, disagreements arose. Jordan visited Mercy to investigate whether the benefits were commensurate with the expenditure on residential facilities and the patients sent by SMC. He concluded that only one-fifth of the charity’s patients came from SMC. If a further grant of 82,000 *yuan*, the amount requested by Lu, was approved, the Council would have covered one-third of Mercy’s total costs.^82^ Lu was therefore asked to provide details of expenditure and plans for future outlay. The Chinese Jiangsu Provincial Authority, in whose district Mercy was located, also had to pay its share. Spending control also affected SMC, as SMC would entirely rely on Mercy to accommodate all its chronic patients.^83^ It is not difficult to see that SMC expected to have the upper hand in spending control. But the competing parties were not going to allow it to take the helm on its own. The emergence of these conflicts was only the beginning of a years-long struggle for control of the administration.

## Business for Economical Confinement

The hospital, similar to what Wayne Soon demonstrated in the development of biomedicine, could be facilitated and constrained by ‘financial support, domestic politics, transnational opposition, and local resistance’.^84^ In Mercy’s case, the focus of the conflict was budgetary control and closely related philanthropic or business positioning. It should be noted that Mercy Hospital did not conform to the British and French model of a private asylum, as it took on the function of the Chinese Charity Hall and absorbed funds from colonial Municipalities and charities.

**Chart 2:**
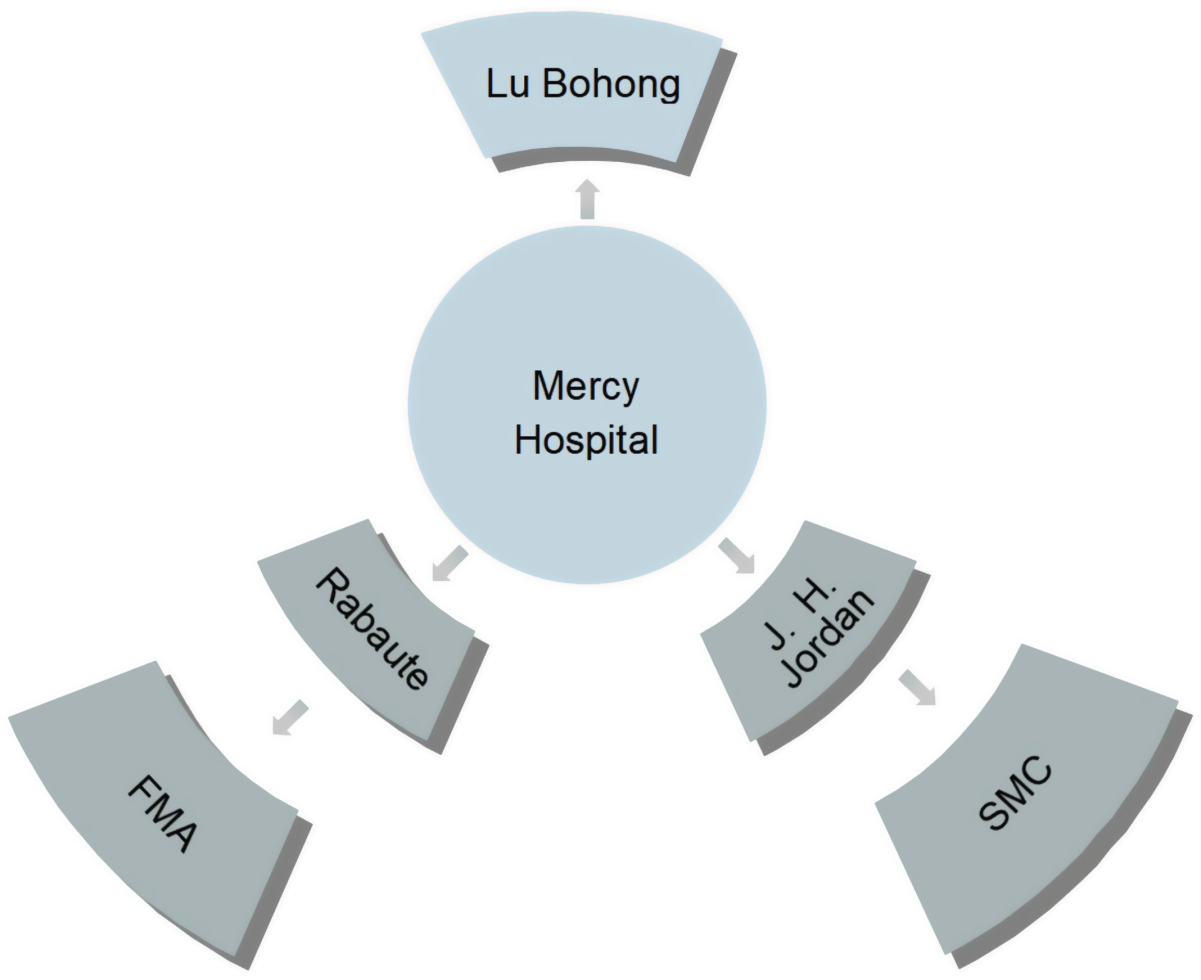
Representatives of management at the Mercy Hospital

Repeated negotiations took place between Lu, on behalf of the local gentry and Catholics, and the SMC with Jordan as his representative, who invited the FMA as a common stakeholder when necessary. Chart 2 shows the tripartite relationships and their representatives in Mercy Hospital. However, as mentioned in the last part, the SMC and the FMA distrusted each other and the FMA developed an ambiguous relationship with Lu. The manipulations, setbacks and compromises with which the Mercy project was fraught make it clear that inter-precinct cooperation was fragile, volatile and prone to individual points of view. Sometimes personal dislike between representatives made a significant difference in cooperation.

Lu and Jordan held opposing values and viewpoints. Lu was committed to fighting for the ‘Chinese’ and local business groups against white supremacy. His philanthropic and religious approach also contradicted the SMC’s business practises. The nationalist identity combined with his forceful personality led him to claim ‘Chinese’ control of the hospital rather than conform to Western patterns and demands.^85^ On the contrary, Jordan had been reluctant to the demands of the local Chinese for a more significant role in municipal administrations and the change in the political environment in China. When Jordan took office in 1930, the Chinese anti-imperial movements had made progress in many areas. Nevertheless, Jordan looked down on his Chinese counterpart. He refused to promote Chinese in municipal service, to work with Chinese or to attend meetings chaired by a Chinese.^86^ When the SMC asked to use a site in Greater Shanghai for human waste disposal and the Chinese authority demanded concessions on detaining students for an exchange, Jordan refused to sacrifice public health for political purposes.^87^ He thought Chinese negotiations as disrespectful to the SMC. The Council also found it difficult to work with the newly assertive and ambitious Chinese authorities. But the ‘foreign firms in China were following a “localisation” strategy to assuage nationalist demands and pressure was growing on the SMC to follow suit’.^88^

SMC sparked controversy with a brand-new administrative proposal on 14 February 1936, after it approved a second grant.^89^ The plan guaranteed Mercy the exclusive position of staff member but under broad control. Mercy was to be run under joint control of the Finance Department, Public Works Department, Public Health Department, Fire Brigade and Police Department to ensure it satisfied standards. The police handled the transfer of the chronically mentally ill from the Settlement to Mercy. In an effort to ensure his right of supervision and adequate representation of SMC, Jordan made his most important proposal on 30 January 1936, which was to appoint a Business Manager with sufficient knowledge to run a hospital. Further grants were to be made only if a Board of Governors composed of laymen and physicians was formed on which the Council would be adequately represented.

In addition to improving SMC oversight, the hiring described above aimed to steer Mercy away from Lu’s philanthropic approach. Jordan personally disliked Lu’s interference in the management of the hospital. In a letter to the SMC Board, he suggested that Lu be given a notice that:

(The) Director or Patron of Hospitals does not normally in other countries give orders and generally interfere with the management of patients in the institutions of which he is a patron ... (Lu) spent far too much of his time at the Hospital.^90^

He disqualified Lu and his Chinese allies for their inexperience and often complete lack of experience in running a modern hospital. Although he had no training in running modern hospitals, Lu was praised at the inauguration ceremony for his 20 years of experience in charity.^91^ Even in areas where the Chinese were qualified, as in the recruitment of the deputy commissioner for the Public Health Department, Jordan chose the British.^92^ Moreover, he and the SMC had been reluctant to provide philanthropic support to the needy, especially the Chinese. ‘The Council’s motivation for attempting to improve the public health of Chinese in the Settlement was in large part for the benefit this brought to the foreign community by removing potential sources of infection’.^93^

Defining Mercy as ‘unbusinesslike’ served to justify SMC interference and to get the French to formulate a common front. Jordan, for example, spared no effort to point out that Mercy was unable to cater for sufficient paying patients and meet modern standards because it was ‘impractically run in a philanthropic rather than business way’.^94^ Moreover, the organisation of a board of directors depended on those in power, be they Chinese or foreigners.

Lu prepared three versions of a plan for the Board of Governors, and received different objections from the SMC each time. The first draft sent to the SMC on 11 March 1936, provided for nine members, one each from the SMC, the FMA and the Shanghai City Government, two from the Catholic Church and four from the local gentry.^95^ For Lu, agreeing to establish of a Board was a compromise because it diminished his power. Thus, his first plan came with the expectation of another annual grant as a reward. Jordan, however, was completely dissatisfied with this proposal, in which SMC provided only represented one-ninth of Board members but contributed one-third of funds. He also complained about the overrepresentation of Catholics, especially local gentry who ‘contribute nothing (financially)’ apart from some experience in running hospitals like Mercy.^96^ He therefore suggested an expert in hospital management as chairman of the Board.

The SMC’s rejection was announced the next day. Rather than respond to the local nobility’s objection, Lu drew the French in with the message that he had received excellent financial support from the FMA and expected further funding from the SMC. The FMA decided not only on an additional building allowance of 41,000 *yuan*, but also a maintenance payment of 5,000 *yuan* in 1936 and an annual grant of 10,000 *yuan* from 1937. Lu felt comfortable reminding Jordan of the increasing number of patients sent by the SMC.^97^ The latter expected a higher contribution from FMA, but Jordan doubted Lu’s information and was reluctant to be put on the spot. He decided to test French’s sincerity himself, expecting to force Lu to listen to the Municipalities but not vice versa.^98^

The favour of the French thus became a decisive factor in the three-sided game, but Jordan was confident of allying himself with them. In his opinion, the French director of Public Hygiene, Rabaute, was ‘a very forcible chap’ whose ‘French logic and directness of speech’ made him a good ally.^99^ Jordan therefore made two proposals to the French: One was to appoint a Business Executive with sufficient knowledge of hospital administration to assist the Superintendent, and the other was to insist on full representation on the hospital’s board of directors. The disadvantages of leaving the hospital in ‘Chinese hands’ were particularly emphasised in his letter in order to gain sympathy for the alliance, which had difficulty sending poorer foreigners to Mercy and its ‘efficient’ ability to cure and discharge patients back to the city posed a threat to the Municipal administrations.^100^ In a word, the original goal of funding Mercy, to remove patients from the Concessions would have been in vain. Therefore, negotiations were to strengthen the cooperation between the two communities, for example, by reallocating the numerous expenditures for certain decorative measures to necessary medical supplies, equipment and instruments. As a result, both Municipalities agreed to forego representation of the local gentry and instead nominate two members of the SMC and Shanghai City Government, and one member each of the FMA and the Catholic Church.^101^

The financial instruments have efficiently achieved the goal of leaving out the Chinese. On 16th April, the Council notified Lu that it would suspend further capital or income grants, except for a grant of 3,000 *yuan* for the current year, which was equivalent to 6 per cent interest on a principal amount of 50,000.^102^ Two months later, Lu responded with a second plan that included 11 members: two each from SMC, FMA, the Chinese Municipality and the Catholic Action Society; the other three would be the project sponsor (Lu himself), the Chief Physician (Fanny Halpern) and the hospital’s priest, who would be well-versed in hospital administration.^103^ In addition, a medical committee would be formed with seven members: one each from SMC, FMA, Chinese City Government and Catholic Action Society as well as the president, the priest and the Chief Physician. This plan seemed to reasonably exclude the Chinese and include hospital and medical specialists.

The SMC, however, was again unhappy with the greater proportion of French interests on the Board. Apart from the fact that a smaller proportion of patients and funding came from the French Settlement, both Lu and the Catholic Action Society (with Lu as its leader) were closely associated with the FMA. So while he agreed with the SMC’s adequate representation and the establishment of a medical committee, Jordan questioned the overrepresentation of Catholic interest, since they contributed almost no money.^104^ He suggests that the Board remain nonsectarian and that the FMA and Catholic Action Society representatives be reduced to one, excluding the priest.^105^

Behind the negotiations was the irreconcilable competition for complete control between the Chinese and SMC. Jordan believed that Mercy’s fame as a philanthropic organisation derived from Municipal subsidies, particularly from SMC, and concluded that the Municipalities should be in charge.^106^ While the second plan reduced Chinese representation, SMC felt that Lu could manipulate the appointment and function of the committees. With the exception of the SMC representative for Jordan, all other members of the Medical Advisory Committee were aligned with Lu’s interests. On 12 September 1936, the SMC’s Deputy Secretary urged Lu to form a separate committee that could not issue directives but could provide aftercare for inmates and extras on holidays or other occasions so that ‘good’ administrative control would not be compromised.^107^ Lu then pointed out the importance of local Chinese to Mercy’s construction and the need to invite them to join the Board of Directors in return for financial support.^108^

The negotiations reached an impasse because no one wanted to give in. The SMC disagreed internally on how to deal with this dilemma. Some suggested obtaining written approval from Lu of the Council’s proposals for the formation of the Board of Governors and the Medical Advisory Committee.^109^ Others believed such action would only increase Lu’s resistance.^110^ The effect of the financial pressure exerted earlier was in question, as the SMC was unwilling to make the situation worse. Finally, it was agreed to inform Mr Lu verbally ‘in any tactful manner’ that the funding would be discontinued, rather than sending official letters.^111^

Lu grew tired of the SMC’s complaints and demands and decided to exercise his absolute control over Mercy Hospital rather than yield to the warning. In his third plan for the Board of Directors, which he sent to Jordan on 28 November, Lu nominated himself chairman with the right to:

Nominate and select all other representatives.

Invite local philanthropists as additional members for the sake of funding.

Appoint the Superintendent.

Be a member of and invite others to join the Medical Advisory Community.

Convene meetings for the Board of Brothers, Board of Directors, and Medical Advisory Community.

Decide on hospital affairs and carry out the decisions of the Medical Advisory Community.^112^

The foreign ally failed to counter Lu’s strong countermovement. The SMC insisted that the true founders were those who provided 80 per cent of the funding, namely the three Municipalities.^113^ A clearly worded notice was given that if the Council’s proposals were not agreed to without unnecessary changes, the funding would cease immediately. Before acting, Jordan wrote to Ralaute to obtain his approval.^114^ However, the French response, however, only made SMC’s situation worse by pointing out that Lu was ‘a very prominent member’ of the FMA, which preferred to ‘proceed independently’.^115^ This attitude was not unexpected, as the French had always been sluggish on municipal services. The French believed that efficient administration was tough, if not impossible.^116^

The see-saw game ended with Lu’s victory. He and his son took the roles of President and Vice-President. The other nine members came from three Municipalities, the Catholic Action Society (two each) and a Medical Director. Lu also chaired the Medical Advisory Committee, which included one representative from each of the above authorities and another from the National Shanghai Medical College.^117^ Undoubtedly, SMC continued to complain, but Lu turned a deaf ear. At the First Meeting of the Medical Advisory Committee, he again pointed out the crucial role he and his son (Lu Ying-geng 陆英耕) played in pushing the project forward and raising funds. If they did not take over the management of the hospital, it would ‘undoubtedly be a loss of face’.^118^ It also followed a verbal threat that he would wash his hands of the matter if the board of directors was composed as proposed by the SMC.

Lu’s insistence on Chinese control and the inclusion of local gentry on the Board is better understood in the context of the anti-imperialist movement. It was not wise for the colonisers in the Republic of China to neglect local power. Working with foreigners posed a significant risk to social prestige, not to mention working under the colonisers’ command. The risk increased after the Japanese occupied Shanghai when Lu was forced to restructure the ruined power and lighting plant and join the Shanghai Citizens’ Association, a pro-Japanese group, to protect his businesses and charities. On 30 December 1937, the morning after he accepted the Japanese terms, Lu was shot on his doorstep by two men disguised as orange vendors.^119^ Rumours made the rounds. Suspicion of being a Japanese collaborator was undoubtedly the main reason. Some said, ‘Chiang kai-shek ordered the assassination, but Shanghai was full of thugs and bandits of all kinds, and French police were open to corruption’.^120^ Many other prominent figures were murdered at the same time. The authorities failed to catch the culprit, and so the case remained unsolved. But this incident led to the Mercy Hospital later being taken over by the foreign administration.

## A Burden to Be Taken Over

The Sino-Japanese War in 1937 changed the fate of China, foreign interests in Shanghai and Mercy Hospital. After the Nationalist government withdrew from eastern China, the Concessional authorities were forced to cede their priorities to Japan. Local hostilities in Shanghai caused severe property damage and created financial difficulties for Mercy Hospital, as access roads and supplies to the hospital were interrupted, and permission from Japanese authorities was required for each visit.^121^ Diseases related to malnutrition could occur at any time; according to Jordan’s report, there was no actual administration, especially after Lu’s death, and no medical assistance except for the German Brothers and the American Sisters.

Without the subscriptions of the Chinese municipalities and gentry, financial grievances soon arose. Neither the SMC nor the FMA were willing to share the Chinese portion. Patients treated without charge increasingly became an eyesore. By early 1938, Mercy sheltered about 300 patients, most of whom were poor and free of charge.^122^ It also began to accept refugees and wounded soldiers from camps and surrounding counties.^123^ Monthly expenses were about $3,000, with a substantial amount spent on refugees.^124^ The SMC and FMA provided limited funds to prevent large numbers of mental patients from being set free.^125^ However, the number fell far short of expenditures. The situation worsened in 1940 as more subscriptions were withdrawn, the number of refugees continued to increase, prices skyrocketed and the deficit reached $26,443.42. At the end of the year, the total number of patients was 336: 66 from the SMC, 92 from the FMA, 36 from Suzhou, a district near Shanghai, and another 142.^126^

SMC promoted discharge of mild patients and operated Mercy most economically.^127^ J. Verdier, who took over as superintendent on 30 June 1938, considered this measure ineffective because most patients were homeless, friendless or offending prisoners.^128^ Releasing them back onto the streets could be more costly and dangerous, as many would find their way to the French Police and then be sent back.^129^ However, the SMC chose to sacrifice public order in order to save money in the turmoil of the crisis. By March 1941, there were only 110 patients left at Mercy.^130^

The SMC also urged Verdier to do everything he could to force the Chinese authorities to take their share, with the expectation that Verdier himself would raise money from charities.^131^ The Shanghai Special Municipal Government eventually contributed $10,000.^132^ For the rest of the deficit, FMA proposed to share it equally with SMC. SMC requested that the deficit be split according to the number of patients, resulting in a 35:20 ratio for FMA and SMC.^133^ It also asked the FMA to collectively require the Chinese to make future payments. The evisions of the FMA and the SMC led to Verdier’s resignation because the ‘municipality adopt a monetary advance repayable by its own payments for the hospitalisation of its patients which is nothing more than covering a deficit by creating another’.^134^ He refused to work in such a difficult environment with a $41,000 deficit.

Both Councils acted quickly to retain Verdier, praising his management ability. More importantly, they soon agreed to form an Advisory Committee to estimate the budget and operating costs and revenues.^135^ This committee replaced in 1941 the Board of Governors appointed by Lu, which was criticised as cumbersome and impractical for its small financial contribution. This measure was taken to avoid laborious negotiations with the founders. Two foreign Municipalities agreed to share the deficit while the Chinese also pledged 20,000 *yuan* for the deficit and future costs for patients from Chinese regions.^136^ The hospital stopped accepting patients who were not paid by the SMC and FMA. By 29 September 1942, Mercy accommodated 378 patients with 50 additional beds.^137^

The residence qualification was applied to divide liability among the three Municipalities to avoid disputes caused by border crossers.^138^ This applied to cases of leprosy, cholera and other infectious diseases. For those who moved across borders, their previous residency status was valid for an additional 6 months if the application was in process in a new area. For cases that moved between regions and were difficult to identify, the three Municipalities would divide responsibility based on where they had lived in the previous 6 months.^139^

On 26 June 1943, Mercy stopped accepting patients, especially females, because there was no room to house them.^140^ The food problem became more serious as prices rose and communities were late in paying the bills. The hospital had overdrawn 346,850 *yuan* with at Catholic Mission. In May 1945, the families of all patients were informed of their return to their care.^141^ Verdier resigned on 1 June 1945, and 159 homeless patients remained until July.^142^ Dong Zaixiu (董在修) thereafter helped maintain.^143^

Mercy Hospital was taken over by the South China Health Department (*Huanan weishengbu*华南卫生部) as a missionary organisation and institution of imperialism in July 1951 and transferred to a ‘Hospital of the People’ under central economic and political planning.^144^ Its staff was asked to obey new ideologies and, in 1954, it was renamed the Shanghai Municipal Psychiatric Hospital (*Shanghai shili jingshenbing yuan*上海市立精神病医院).^145^ Four years later, the psychiatric faculties of Shanghai First and Second Medical College and all other mental hospitals were amalgamated into the Shanghai Psychiatric Hospital (*Shanghai shi jingshenbing fangzhi yuan*上海市精神病防治院), a centre to provide preventive care along with advertising, teaching and research functions. From the 1980s, it pioneered a series of transnational projects as well as the WHO Collaborating Centre for Research and Training in Mental Health.^146^ Currently, the Shanghai Mental Health Centre, also known as the Shanghai Psychological Consultative Centre, still leads psychiatric research and treatment in China.

## Conclusion

This study examined the transnational politicking and medical financing of the little-studied Mercy Hospital in semi-colonial Shanghai. It shifted from the dominant account of institutionalisation to a behind-the-scenes analysis, shedding light on the sponsors who vied for control of the hospital and were driven by competing aims. By revealing their protracted negotiations, this study argues against a straightforward transfer of biomedicine from the West to the East.^147^ I urge attention to local factors and the actors’ quest to adapt Western science to modern China.^148^

The history of Mercy Hospital reveals several key features of medical modernisation in Republican China, the most notable of which were nationalistic and symbolic. Mercy Hospital was initiated in response to numerous reform demands to strengthen the nation. Indeed, newspapers frequently emphasised that it was Chinese-owned. Its size and state-of-the-art equipment served more to equate Shanghai’s modernity with Western metropolises like Paris than to guarantee excellent medical care. However, asylums were the last resort for both foreigners and locals in the Republican period due to economic considerations and social or racial discrimination.

The hospital’s transnational governance led to serious conflicts because Lu’s philanthropic path fundamentally contradicted SMC’s business orientation. Lu aspired to be a laudable philanthropic and religious enterprise with maximum investment to benefit the mostly Chinese needy. On the other hand, the colonial authorities funded Mercy Hospital to shake off troublesome Chinese refugees, lock up the mad foreigners and maintain order on the streets and white supremacy. In this way, they pushed for cost-effective accommodation of the smallest possible number of needy foreign patients. At the heart of the long-running financial disputes was the right to control, an even investment ratio and low costs. Neither orientation took improving treatments as a top priority. Halpern hoped ‘the Mercy will eventually form a centre for the study and teaching of mental diseases for the whole of this part of China’.^149^ However, she had little influence on hospital management. Scientific standards were mainly used to justify racial segmentation, with financial instruments ensuring implementation.

The challenge to funding Mercy Hospital contrasts with two other forms of transnational cooperation in Republican China. PUMC shared with Mercy Hospital transnational governance, large foreign endowments and ongoing budgetary disputes. But the Rockefeller Foundation secured complete control of it. The conflicts between the Chinese and foreign staff were more about the localisation of training and diversification of the teaching staff than about the struggle for Chinese ownership. By comparison, cooperation among indigenous and overseas Chinese could be more nationalistic, even shunning foreign intervention by relying solely on overseas Chinese money. A typical example was the failure of Lim Boon Keng’s (1869–1957) repeated attempts to fund a medical school at Xiamen University because his patron Tan Kah Kee (1874–1961) was determined to accept donations exclusively from the Chinese diaspora.^150^ Comparatively, funds from America and scientific support from around the world helped develop biology and marine sciences at the university. These multiple mechanisms of cooperation between the Chinese and the colonisers, the missionaries and the diaspora encourage further research into colonial medical practice and the modernisation of China.

Transnational collaboration and dissent in the management of Mercy Hospital added new facets to the complicated relationship between colonial medicine and nationalist medicine from both macro and micro perspectives. Lu fought for more Chinese rights in the Council, not just control of the hospital. The SMC tried to maintain its colonial rule by minimising the rights and benefits of the Chinese. This oppression led to radical resistance when the British tried to remove Lu from the board. This game reflected the burgeoning conflicts across the country sparked by Chinese anti-imperial movements and colonial oppression. On an individual level, the British may have been ashamed to submit to the natives. Lu and the local Chinese nobility thought similarly in their fear of being labelled collaborators. Lu’s assassination was not inconsistent with his close relations with the foreign authorities. The collaboration ended with the British and French quickly forging a new alliance and removing the Chinese from the board and the wards until the communist regime took over the hospital.

## Footnotes

1 Richard Keller, ‘Madness and Colonization: Psychiatry in the British and French Empires, 1800–1962’, *Journal of Social History*, 2001, 35, 295–326; Anouska Bhattacharyya, ‘Indian Insanes: Lunacy in the “Native” Asylums of Colonial India, 1858–1912’ (PhD thesis, Harvard University, 2013); Waltraud Ernst, *Mad Tales from the Raj: European Insane in British India, 1800–58* (London: Routledge, 2004); Catharine Coleborne, ‘Families, Patients and Emotions: Asylums for the Insane in Colonial Australia and New Zealand, 1880–1910’, *Social History of Medicine*, 2006, 19, 425–42; Harry Minas and Milton Lewis, eds, *Mental Health in Asia and the Pacific: Historical and Cultural Perspectives* (New York: Springer, 2017); Kam-Shing Yip, ‘A Historical Review of Mental Health Services in Hong Kong (1841 to 1995)’, *International Journal of Social Psychiatry*, 1998, 44, 46–55; Roy Porter and David Wright, eds, *The Confinement of the Insane: International Perspectives, 1800–1965* (Cambridge: Cambridge University Press, 2003).

2 Harry Yi-Jui Wu and Wen-ji Wang, ‘Making and Mapping Psy Sciences in East and Southeast Asia, East Asian’, *Science Technology and Society an International Journal*, 2016, 10, 109–120; Wenjing Li and Heinz-Peter Schmiedebach, ‘German Wine in an American Bottle: The Spread of Modern Psychiatry in China, 1898–1949’, *History of Psychiatry*, 2015, 26, 348–58; Geoffrey Blowers, ‘Bingham Dai, Adolf Storfer, and the Tentative Beginnings of Psychoanalytic Culture in China: 1935–1941’, *Psychoanalysis and History*, 2004, 6, 93–105; Zheng Fei, ‘In the Name of Psy-Disciplines: The Development of Mental Hygiene in Republican China’, in Irina Lyubchenko and Fiona Ann Papps, eds, *Schizo: The Liberatory Potential of Madness* (Leiden: Brill, 2015), 101–11; Nancy Chen, ‘Translating Psychiatry and Mental Health in Twentieth-Century China’, in Lydia H. Liu, ed., *Tokens of Exchange: The Problem of Translation in Global Circulations* (Durham, NC: Duke University Press, 1999), 306; Volker Scheid, ‘Constraint 鬱as a Window on Approaches to Emotion-Related Disorders in East Asian Medicine’, *Culture, Medicine, and Psychiatry*, 2013, 37, 2–7.

3 N. Blum and E. Fee, ‘The First Mental Hospital in China’, *American Journal of Public Health*, 2008, 98, 1593. Peter Paul Szto, ‘The Accommodation of Insanity in Canton, China: 1857–1935’ (PhD thesis, University of Pennsylvania, 2002); Peter Paul Szto, ‘Psychiatric Space and Design Antecedents: The John G. Kerr Refuge for the Insane’, in Howard Chiang, ed., *Psychiatry and Chinese History*, 79–90; L. Fu, ‘Medical Missionaries in China: John Glasgow Kerr (1824–1901) and Cutting for the Stone’, *Journal of Medical Biography*, 2018, 26, 194–202; Hugh Shapiro, ‘The View from a Chinese Asylum: Defining Madness in 1930s Peking’ (PhD thesis, Harvard University, 1995).

4 Zhiying Ma, ‘An Iron Cage of Civilization? Missionary Psychiatry, the Chinese Family and a Colonial Dialect of Enlightenment’, in Chiang, *Psychiatry and Chinese History*, 91–110.

5 Harry Yi‐Jui Wu, ‘From Means to Goal: A History of Mental Health in Hong Kong from 1850 to 1960’, in Harry Minas, ed., *Mental Health in China and the Chinese Diaspora: Historical and Cultural Perspectives* (Cham: Springer International Publishing, 2021), 74.

6 Emily Baum, *The Invention of Madness: State, Society, and the Insane in Modern China* (Chicago: University of Chicago Press, 2018), 117–29.

7 Wayne Soon, *Global Medicine in China: A Diasporic History* (California: Stanford University Press, 2020), 4.

8 Jinping Ma, ‘Remoulding the Chinese Mind: Mental Hygiene Promotion in Republican Shanghai’ (PhD thesis, The University of Warwick, 2019), 83–95.

9 Wen-ji Wang, ‘An International Teamwork: Mental Hygiene in Shanghai during the 1930s and 1940s’, *History of Psychology*, 2019, 22, 289–308.

10 Business is a term repeatedly used in the correspondences of the Shanghai Municipal Council in negotiating the management of Mercy Hospital. It may remind readers of the word ‘trade’ as used in the research of Roy Porter and Marjorie Meehan about private madhouses in late eighteenth-century England when investors with no medical training built houses of confinement managed as businesses; profitability was ensured due to both a lack of competition and concern for public order in the context of medical commercialisation. However, the ‘business’ in the case of Shanghai Mercy Hospital was indicated more by its budget balance than financial speculation. As this article will show, while the two foreign Municipal Councils tried to make the best of their patronage, Mercy Hospital functioned more as a charitable and municipal management institute.

11 Frank Dikötter, *Crime, Punishment and The Prison in China* (London: Hurst, 2002), 46.

12 Iwo Amelung, ed., *Discourses of Weakness in Modern China: Historical Diagnoses of the ‘Sick Man of East Asia’* (Frankfurt: Campus Verlag, 2020).

13 Zuoyue Wang, ‘Saving China through Science: The Science Society of China, Scientific Nationalism, and Civil Society in Republican China’, *Osiris*, 2002, 17, 291–322.

14 Emily Baum, ‘Healthy Minds, Compliant Citizens: The Politics of “Mental Hygiene” in Republican China, 1928–1937’, *Twentieth-Century China*, 2017, 42, 215–33.

15 Vivien Ng, *Madness in Late Imperial China: From Illness to Deviance* (Norman: University of Oklahoma Press, 1990), 66–74.

16 Baum, *The Invention of Madness*, 41.

17 Akihito Suzuki, ‘The State, Family, and the Insane in Japan, 1900–1945’, in Roy Porter and David Wright, eds, *The Confinement of the Insane: International Perspectives, 1800–1965* (Cambridge: Cambridge University Press, 2003), 193–245.

18 ‘Shen Jiaben diaocha riben jianyu qingxing qingdan’ 沈家本调查日本监狱情形清单 (The Investigation of Shen Jiaben on Japanese Prisons), 12 August 1907, *Shen bao*, No. 12405, 10.

19 ‘Shen Jiaben diaocha riben jianyu qingxing qingdan’ 沈家本调查日本监狱情形清单 (The Investigation of Shen Jiaben on Japanese Prisons), 14 August 1907, *Shen bao*, No. 12407, 10.

20 There was a John Kerr Refuge for the Insane in Guangzhou (1898–1937), the Beijing Psychopathic Hospital (established in 1933) and the Wei Hongsheng (魏鸿声) Psychopathic Hospital in Beijing (established in 1934) and a ward in the Gospel Hospital in Suzhou (in operation between 1929 and 1936).

21 Ma, ‘Remoulding the Chinese Mind’, 82–83.

22
*Hongkou Qu Zhi* 虹口区志 (Gazetteers of Hongkou District), volume 31, chapter 5, section 3, ‘Zhuanbing fangzhi’ 专病防治 (Control and Prevention of Specific Diseases), part 3. Available from:
http://www.shtong.gov.cn/Newsite/node2/node4/node2249/node4418/node20226/node24878/node62966/userobject1ai11325.html.

23 Baum, ‘Choosing Cures for Mental Ills’, 18.

24 ‘Xin Puyütang tianjian fengrenyuan’ 新普育堂添建疯人院 (A New Asylum was Established in New Puyütang), 18 March 1914, *Shen bao*, No. 14762, 10; Baum, *The Invention of Madness*, 47.

25 Isabella Jackson, *Shaping Modern Shanghai: Colonialism in China’s Global City* (Cambridge: University of Cambridge Press, 2018), 193.

26 Charles Westbrook, ‘Psychiatry and Mental Hygiene in Shanghai: Historical Sketch’, *The American Journal of Psychiatry*, 1953, 110, 301–6; Shanghai Municipal Library (SML), ‘The Modern Health Campaign in China: An Up-to-Date Hospital for Nervous Diseases’, 30 June 1935, *North-China Daily News*.

27 Jackson, *Shaping Modern Shanghai*, 193.

28 ‘Puci liaoyangyuan zuo kaimu’ 普慈疗养院昨开幕 (Mercy Hospital Opened Yesterday), 30 June 1935, *Shen bao*, No. 22334, 14.

29 ‘Fa zongshiling deng zuo canguan puciyuan’ 法总领事等昨参观普慈院 (The French Consular visited Mercy Hospital Yesterday), *Shen bao*, 11 November 1936, No. 22821, 12; ‘Fa dashi deng canguan puci liaoyangyuan’ 法大使等参观普慈疗养院 (The French Consular Visited Mercy Hospital), 2 December 1936, *Shen bao*, No. 22842, 16. ‘Zhongyang weishengshu yiwu kaochatuan canguan puci liaoyangyuan’ 中央卫生署医务考察团参观普慈疗养院 (The Medical Affairs Investigation Mission Visited Mercy Hospital), 10 April 1937, *Shen bao*, No. 22962, 12.

30 ‘Puci liaoyangyuan dingqi kaimu’ 普慈疗养院定期开幕 (Mercy Hospital is open on Time), 22 June 1935, *Shen bao*, No. 22326, 10.

31 ‘Puci liaoyangyuan jinxun’ 普慈疗养院近讯 (News about Mercy Hospital), 17 November 1935, *Shen bao*, No. 22473, 11; ‘Shanghai puci liaoyangyuan’ 上海普慈疗养院 (Shanghai Mercy Hospital), 8 March 1936, *Shen bao*, No. 22576, 13; ‘Shanghai puci liaoyangyuan jinxun’ 上海普慈疗养院近讯 (News about Shanghai Mercy Hospital), *Shen bao*, 12 July 1936, No. 22700, 17.

32 Baum, ‘Spit, Chains, and Hospital Beds’, 303.

33 The colours of wall and furnishings included red, yellow, blue and black. ‘Mercy Hospital Opened Yesterday’.

34 Richard Zhang, ‘Dr. Fanny Halpern, A Psychiatric Go-between of 1930s Shanghai’, *Hektoen International Journal of Medical Humanities*, 2020, 12. Available from https://hekint.org/2020/05/28/dr-fanny-halpern-a-psychiatric-go-between-of-1930s-shanghai/ (Last read, 21 December 2022).

35 SML, Mental Hospice to be Opened End of June, 1 June 1935, *The China Press*.

36 Baum, ‘Spit, Chains, and Hospital Beds’, 303.

37 Ma, ‘Remoulding the Chinese Mind’, 133.

38 ‘Jingshenbing yuan neimu’ 精神病院内幕 (Inside the Asylum), 5 August 1946, *Shen bao*, No. 24594, 8.

39 ‘Lubohong shi chuangban puci liaoyangyuan jiri kaizhen’ 陆伯鸿氏创办普慈疗养院即日开诊 (The Mercy Hospital founded by Lu Bohong is to Accept Patients), 25 September 1935, *Shen bao*, No. 22421, 12.

40 ‘Beiqiao puci liaoyangyuan zuori zhaodai baojie’ 北桥普慈疗养院昨招待报界 (Mercy Hospital at Beiqiao Convened a Press Conference Yesterday), 8 August 1936, *Shen bao*, No. 22727, 15; ‘Puci liaoyangyuan she nanmin shourongsuo’ 普慈疗养院设难民收容所 (Mercy Hospital Set up a Shelter for the Needy), 24 November 1937, *Shen bao*, No. 23188, 6.

41 ‘Daibiaodahui yuanman bimu’ 代表大会圆满闭幕 (The Congress Successfully Concluded), 16 September 1935, *Shen bao*, No. 22412, 13.

42 Anne-Marie Brady and Douglas Brown, eds, *Foreigners and Foreign Institutions in Republican China* (London: Routledge, 2012), 131.

43 Jackson, *Shaping Modern Shanghai*, 114.

44
*Ibid.*, 6.

45 Robert Bickers, ‘Incubator City: Shanghai and the Crises of Empires’, *Journal of Urban History*, 2012, 38, 862–78.

46 Jackson, *Shaping Modern Shanghai*, 18.

47
*Ibid.*, 192.

48
*Ibid.*

49
*Ibid.*, 5.

50
*Ibid.*, 19.

51
*Ibid.*, 171, 173.

52
*Ibid.*, 214.

53
*Ibid.*, 190.

54
*Ibid.*

55
*Ibid.*, 189.

56
*Ibid.*, 193.

57 Denise Austin, *‘Kingdom-Minded’ People: Christian Identity and the Contributions of Chinese Business Christians* (Leiden: Brill, 2011), 178.

58 D. E. Mungello, *The Catholic Invasion of China: Remaking Chinese Christianity* (Lanham: Rowman & Littlefield, 2015), 52.

59 Austin, *‘Kingdom-Minded’ People*, 172.

60 Steven Pieragastini, ‘Imperium inter Imperia: the Catholic Church in Shanghai, 1842–1957’ (PhD thesis, Brandeis University, 2017), Introduction.

61 Katherine Koch, ‘The Pennsylvania Passionist Who Became Chinese: Fr. Basil Bauer, C.P.’, 35–54. Available from: chrome-extension://oemmndcbldboiebfnladdacbdfmadadm/https://core.ac.uk/download/pdf/234049791.pdf (Last read, 3 June 2021).

62 Available from: http://bdcconline.net/en/stories/lu-bohong (Last read, 23 July 2021).

63 Austin, *‘Kingdom-Minded’ People*, 173.

64 Translation of the letter to the secretary and Chinese co-secretary from Shanghai Hsing Piu Yoh Tong (上海新普育堂), 28 November 1933, U1-16-592, 4, Shanghai Municipal Council Correspondence, Shanghai Municipal Archives, Shanghai (henceforth U1-16-592).

65 Ma, ‘Remoulding the Chinese Mind’, 80–82.

66 Jackson, *Shaping Modern Shanghai*, 104.

67
*Ibid.*, 183–84.

68
*Ibid.*, 174.

69 The Commissioner of Public Works remarks that ‘Chinese plans were always very sketchy and one could do little in influencing them in the matter of the building; they would build what they wanted themselves and no doubt it would come up to their standards and fill their requirements’. U1-16-592, 18.

70 ‘London 30th January, The Asylum Fire’, 12 March 1903, *Reuters*.

71 The disagreement was actually about the necessity of constructing padded cells to segregate violent cases. A Conference with T. K. Ho, Lo Pa Hong, Harpur and the Commissioner of Public Health, 29 January 1934, U1-16-592, 7.

72 Letter to the Secretary from J. H. Jordan, Commissioner of Public Health, 8 October 1936, U38-5-1158, 35.

73 Letter from J. H. Jordan, 8 February 1936, U1-16-592, 100.

74 Extract from Minutes of Health Committee Meeting of 6 February 1934, U1-16-592, 8.

75 The Commissioner of Public Works Remarks, U1-16-592, 18.

76 Letter from Jordan, 30 January 1936, U1-16-592, 93.

77 J. H. Jordan to the Acting Secretary, 11 November 1935, U1-16-592, 57.

78 Yesterday’s Visit to the Hospital, U1-16-592, 53.

79 Letter from Treasurer and Controller J. T. Ford: Mercy Hospital Request for Further Assistance towards the Cost of Building, 9 December 1935, U1-16-592, 70.

80 The continuing grant was ‘based on the average percentage bed occupancy during the three years ending July 1933’, but ‘will require consideration’ on the completion of the Mercy Hospital. Letter from Acting Treasurer and Controller A. W. Macphail, 27 September 1934, U1-16-592, 54.

81 In his letter Lu wrote that ‘Mayor Wu has kindly promised me a grant from the City Government of Shanghai, although the exact amount has not been decided. The French Municipal promised to give the Hospital a sum of 41,000 as a further grant for the building and installation expenses and another 10,000 every year as yearly maintenance expenses. Out of the 41,000 and 35,000 was advanced on the 21st of January on account of the financial difficulty which the hospital has met with at the end of the lunar year’. Letter from Lu Bohong, 31 January 1936, U1-16-592, 96.

82 Letter from Lu Bohong: Mercy Request Further Contribution, 8 November 1935, U1-16-592, 55.

83 At the Joint Finance and Health Committee meeting of March 19, 1935, the Council decided that the ‘future grant assistance for mental cases would be limited to the Mercy Hospital alone and would not be spread over several private mental hospitals, thus avoiding duplication’. Memorandum to Health and Finance Committee, Care of the Insane, 14 February 1936, U1-16-592, 103.

84 Soon, *Global Medicine in China*, 3.

85 Sherman Cochran, *Chinese Medicine Men: Consumer Culture in China and Southeast Asia* (Cambridge: Harvard University Press, 2006), 162.

86 Jackson, *Shaping Modern Shanghai*, 104, 197.

87
*Ibid.*, 196.

88
*Ibid.*, 104.

89 Letter from Ford, 22 January 1936, U1-16-592, 88.

90 Letter to the Secretary from J. H. Jordan, Commissioner of Public Health, 8 October 1936, U38-5-1158, 35.

91 ‘Mercy Hospital Opened Yesterday’.

92 Jackson, *Shaping Modern Shanghai*, 104.

93
*Ibid.*, 186.

94 Jordan to Rabaute, 23 March 1936, U38-5-1158.

95 Summary of Early Correspondence, U1-16-591, 3.

96 Summary of Early Correspondence, 3. Letter from Jordan, U1-16-592, 16 March 1936, 116.

97 In his letter, Lu reminded that ‘the number of patients from the Settlement admitted to this hospital is over 50, it is quite possible that this number will increase year by year’. Translation of Letter from the Mercy Hospital, 17 March 1936, U1-16-592, 118.

98 J. R. Jones agreed with Jordan that the proposals of Lu were quite unsatisfactory. He suggested that ‘it might be as well to know to what extent the French Health authorities concur. I think it would be much more effective to ask Mr. Lo to consider our suggestion as to management than to ask for further suggestion from him’. Secretary J. R. Jones to Jordan, 16 March 1936, U1-16-591, 4.

99 Jordan to Jones, 20 March 1936, U1-16-591, 5.

100 Jordan to Rabaute, 23 March 1936, U38-5-1158.

101 Mercy Hospital: Views of French Public Health Administration, 1 April 1936, U1-16-591, 14.

102 Letter to Lu, 16 April 1936, U1-16-592, 123.

103 Translation of Letter from the Mercy Hospital, 3 June 1936, U1-16-592, 125.

104 Jordan thought that the hospital priest was ‘a concession to the religious nature of the enterprise’, but this resulted in raising ‘the representation of the Catholic Society to four, and thus practically giving them the casting vote’. Letter from Jordan, 8 June 1936, U1-16-592, 125.

105 Memorandum for Health Committee, 8 July 1936, U1-16-591, 20–22. The final suggestion: (a) the Board should consist of seven members: two from the SMC, two from the Chinese Municipality, one from the FMA, one from the Catholic Action Society and one being the Hospital’s president; (b) a separate Medical committee of five be formed to act in a consultative capacity only: one from SMC, one from the Shanghai City Government, one from the FMA, one of the Catholic Action Society and the Chief physician of the Hospital.

106 Jordan confessed that he had ‘little sympathy with charitable organizations whose charity is subsidized through Municipal funds and fail to understand how they get the name of charitable organizations’. Letter from Jordan, 11 September 1936, U1-16-591, 26.

107 Deputy Secretary to Lu, 12 September 1936, U1-16-591, 27.

108 Lu thought ‘the Mercy was organized through the aid of leading members of the local gentry, we deem it advisable to invite as many charity workers to serve on our Board of Directors as possible so that financial assistance may be expected from them’. Letter from Lu, 9 September 1936, U1-16-591, 25.

109 Reply of the Chairman of Public Health to Jordan about Mercy Hospital. U1-16-591, 38.

110 Letter from Jordan, 30 October 1936, U1-16-591, 40.

111 Secretary to Jordan, 2 November 1936, U1-16-591, 41.

112 Constitution of the Board of Directors of the Shanghai Mercy Hospital, 28 November 1936, U1-16-591, 46.

113 Letter from Jordan, 9 December 1936, U1-16-591, 50.

114 Jordan to Ralaute, 14 December 1936, U1-16-591, 55.

115 30 December 1936, U1-16-591, 57–58.

116 ‘They (the French) are not anxious to take too much responsibility for its administration, as they take the view that it would be difficult if not impossible to control the administration effectively’. Extract from Council Minutes, 20 January 1937, U1-16-591, 60.

117 List of the Board of Governors of Shanghai Mercy Hospital, U1-16-591, 92.

118 Report of the Meeting with Directors of the Hospital, 31 May 1937, U1-16-591, 99.

119 David Strong, *A Call to Mission: A History of the Jesuits in China 1842-1954* (Washington, DC: ATF Press, 2018), 280.

120 Parks M. Coble, *Chinese Capitalists in Japan’s New Order: The Occupied Lower Yangzi, 1937–1945* (Berkeley, CA: University of California Press, 2003), 71.

121 9 February 1938, U1-16-591, 116.

122 Letter from Loh Ying-keng, Vice-president of the Shanghai Mercy Hospital, 14 January 1938, U1-16-592, 157.

123 Austin, *‘Kingdom-Minded’ People*, 181.

124 Letter from Jordan, 14 February 1938, U1-16-591, 18.

125 Letter from Jordan, 11 May 1938, U1-16-591, 131.

126 Special Committee and Meeting of Grant-in-aid, 8 July 1941, U1-16-591, 216.

127 Jordan to Y. Falud, Director of Public Hygiene and Assistance, 26 July 1940, U1-16-591, 163.

128 Father J. Verdier of the Catholic Mission started to handle the administration of the hospital due to Japanese war. 12 July 1938, U1-16-591, 138. Another Catholic Brother Rocane Eraun was in office from February to June 1938. Letter from Lo Yin Kung, U1-16-591, 126.

129 Letter from Father Verdier (Mission du Kiang-nan, Catholic) to Jordan, August 1940, U1-16-591, 170.

130 Jordan to the Secretary and Commissioner General, 20 March 1941, U1-16-591, 205.

131 Letter from the Secretary and Commissioner General, 27 March 1941, U1-16-591, 208.

132 Letter from A. B. Lang, Assistant Secretary to the Acting Treasurer, 27 June 1941, U1-16-591, 214.

133 Special Committee and Meeting of Grant-in-aid, 8 July 1941, U1-16-591, 216.

134 Translation of Letter from Verdier to FMA, 1 September 1941, U1-16-591, 233.

135 Extract from Coordinating Committee Minutes, 8 April 1943, U1-16-591, 345.

136 Translation of Letter from the Treasurer of the FMA to E. P. Oliver, Acting Treasurer of SMC, 11 September 1941, U1-16-591, 239. U1-16-591, 271, 312.

137 Letter from the Superintendent of the Hospital to Jordan, 12 October 1942, U1-16-591, 304.

138 Memorandum for Inter-municipal Conference, 9 November 1942, U1-16-591, 319.

139 Inter-municipal Conference, 19 November 1942, U1-16-591, 323.

140 Verdier to Jordan, 26 June 1943, U1-16-591, 352.

141 Verdier to Jordan, U1-16-591, 479.

142 Resignation Letter from Verdier, U1-16-591, 478. U1-16-591, 500.

143 U1-16-591, 479.

144 B242-1-477-90, 47. Xun Zhou, *The People’s Health: Health Intervention and Delivery in Mao’s China, 1949–1983* (Montreal: McGill-Queen’s University Press, 2020), 257.

145 Q580-16-12, 13.

146 B242-4-856-70, 77.

147 Soon, *Global Medicine in China*, 12.

148 Chiang, *Psychiatry and Chinese History*, 15.

149 Report on Mercy Hospital by Superintendent of Hospitals, 17 April 1936, U1-16-591, 143.

150 Soon, *Global Medicine in China*, 41.

